# Effect of Controlling Nutritional Status Score (CONUT) and Prognostic Nutritional Index (PNI) on patients after spinal tuberculosis surgery

**DOI:** 10.1038/s41598-022-19345-8

**Published:** 2022-09-26

**Authors:** Long-Yao Cao, Si Cheng, Lu Lin, Ming-Xin Chen

**Affiliations:** Department of Orthopedic Surgery, The Second Affiliated Hospital of Chongqing Medical University, No.76, Linjiang Road, Yuzhong District, Chongqing, 400010 China

**Keywords:** Biomarkers, Medical research, Risk factors

## Abstract

The controlling nutritional status (CONUT) score and prognostic nutrition index (PNI) are immune-nutritional biomarkers that are related to clinical prognosis. Previous studies have reported using them to predict the prognosis of traumatic brain injury, tumours and other diseases. The purpose of this study was to evaluate the relationship between the PNI and CONUT score and the one-year prognosis of patients with spinal tuberculosis (STB). In this study, the clinical characteristics of 97 patients with STB who underwent debridement and internal fixation at our institution between 2015 and 2020 were retrospectively analysed. According to the receiver operating characteristic (ROC) curve, patients were divided into two groups: a high CONUT group and a low CONUT group. Patients were also divided into a high PNI group and a low PNI group. One-year postoperative prognosis was evaluated by the clinical cure standard. Patients in the favourable group were younger and had a lower rate of pneumonia and urinary tract infection, higher PNI and lower CONUT score than those in the favourable group (P < 0.05). There was an obvious correlation between the PNI and CONUT score (r = − 0.884, P < 0.05). The areas under the curve (AUCs) of the CONUT score and PNI for predicting unfavourable prognosis were 0.888 (95% CI 0.808–0.943, P < 0.001) and 0.896 (95% CI 0.818–0.949, P < 0.001), respectively. The adjusted odds ratios (ORs) of the CONUT score and PNI for predicting unfavourable outcomes were 2.447 (95% CI 1.518–4.043, P < 0.001) and 0.689 (95% CI 0.563–0.843, P < 0.001), respectively. Higher CONUT scores and a lower PNI were associated with adverse outcomes in patients with spinal tuberculosis, and the CONUT score and PNI might be independent predictors of adverse outcomes of spinal tuberculosis postoperatively.

## Introduction

Tuberculosis is one of the most established infections in the world, tracing all the way back to Egyptian mummies in 3400 BC^1^. Skeletal muscle tuberculosis is seen in almost 10% of patients with dynamic tuberculosis. The spine is the most widely recognized site of skeletal tuberculosis (50% of patients with skeletal tuberculosis)^2,3^. As spinal tuberculosis is a chronic infectious disease, the time from onset to diagnosis may vary from weeks to years, and the onset is occult. The clinical manifestations may be only back pain, with or without systemic symptoms. Percival Pott^4,5^ previously concluded that the associated paraplegia brought about by the destruction of the front vertebral section and the development of kyphosis is one of the most dangerous pathological changes in the musculoskeletal system. Surgical treatment includes drainage of the abscess, lesion clearance and fusion and internal fixation, with the purpose of reconstructing spinal stability. Spinal tuberculosis is not common in developed countries, and most patients are migrants from tuberculosis-epidemic countries. Management and treatment of spinal tuberculosis have become more challenging with global migration and drug resistance. Pulmonary tuberculosis is the most common form of tuberculosis, and many studies have focused on the treatment of pulmonary manifestations. However, less treatment and management related to spinal tuberculosis is one of the reasons for poor prognosis due to delayed diagnosis or improper management. The anticipation of medical procedures for spinal tuberculosis is affected by many variables, among which tuberculosis disease control and nourishing status are two key elements^6–9^. Therefore, it is very important to use effective and convenient nutrition-related biomarkers to evaluate patients at admission for predicting potential adverse prognosis.

Previous studies have recommended the use of various indicators to predict the prognosis of spinal tuberculosis after surgery, but they are limited to the results of a single laboratory examination, and the prognostic evaluation of spinal tuberculosis after surgery is not comprehensive^10–12^. Therefore, the utilization of perioperative nutritional status to predict surgical prognosis and adverse outcomes of spinal tuberculosis needs to be further established. The prognostic nutritional index (PNI), based on serum albumin and peripheral blood lymphocyte counts, was originally used to assess preoperative nutritional status, surgical risk, and postoperative complications in surgical patients. It has been proven to be a prognostic biomarker of various solid tumours, acute heart failure, posttraumatic brain injury and coronary artery disease^13^. A promising new immunonutrition score, controlling nutritional status (CONUT), is an early screening tool for malnutrition because in addition to albumin and lymphocyte counts, the CONUT score also includes cholesterol levels^14^. The PNI and CONUT score are objective and simple indicators for evaluating the nutritional status of the body. However, the correlation between the PNI, CONUT score and surgical outcomes in patients with spinal tuberculosis has not been reported. The aim of this study was to evaluate whether the perioperative PNI and CONUT can be used as effective biomarkers for predicting prognosis in patients undergoing spinal tuberculosis surgery.

## Methods

### Study design and selection of patients

This study was approved by the ethics committee of the Second Affiliated Hospital of Chongqing Medical University. All our research methods are in accordance with the Helsinki Declaration and relevant guidelines / regulations and all participants informed consent to the study. Informed consent was obtained from all participants in the study. In this retrospective, observational, single-centre study, we retrospectively analysed the clinical characteristics of patients with spinal tuberculosis hospitalized in the Spinal Surgery Department of the Second Affiliated Hospital of Chongqing Medical University from February 2015 to December 2020. The inclusion criteria were as follows: (1) adults diagnosed with active spinal tuberculosis confirmed by radiography, histopathology and bacteriology^15^; (2) all patients had surgical indications and received primary debridement and bone graft fusion internal fixation for spinal tuberculosis; (3) antituberculous drugs were regularly used for 2–4 weeks before surgery, when ESR < 50 mm/1 h or C-reactive protein (CRP) < 30 mg/L; and (4) blood was drawn regularly for laboratory testing before and after surgery. In addition, the exclusion criteria were as follows: (1) patients who received immunomodulatory treatment before admission, including biological agents, azathioprine, corticosteroids, and methotrexate; (2) patients who underwent spinal debridement surgery; (3) incomplete laboratory testing; (4) patients who refused surgical intervention; (5) lesion specimens that were proven to be pyogenic infection caused by other bacteria; and (6) diagnosis of other diseases related to malnutrition, such as chronic diarrhoea, hepatic sclerosis, and malabsorption syndrome, and other diseases related to inflammation and immunity, such as rheumatoid arthritis, ankylosing spondylitis, and lupus erythematosus. A total of 125 patients were included in this study, in which 1 patient was underage, 4 patients who had undergone debridement and bone graft fusion internal fixation before admission, 8 patients with incomplete clinical data, 2 patients who had not undergone debridement and bone graft fusion internal fixation, and 13 patients who were lost to follow-up. Finally, 97 patients were included.

### Methods

We retrospectively assessed the history and clinical characteristics of the patients as prognostic factors, including age, sex, body mass index (BMI), clinical history of hypertension, diabetes mellitus, and cardiovascular disease. In addition, lifestyle risk factors, including smoking and drinking, and postoperative complications, such as incision infection, deep vein thrombosis and pneumonia, were recorded. We also collected preoperative laboratory biomarkers, including serum albumin, cholesterol, and total lymphoid count. PNI was calculated using the following formula: 10 × serum albumin (g/dL) + 0.005 × total lymphocyte count (/mm^3^). The serum albumin concentration, total peripheral lymphocyte count, and serum total cholesterol concentration were used to calculate the CONUT score (Table 1). According to the receiver operating characteristic (ROC) curve, the best cut-off PNI was 38.6, and the best cut-off CONUT score was 5. Spinal tuberculosis patients were divided into two groups: a high CONUT group (> 5) and a low CONUT group (≤ 5). Patients with PNI > 38.6 and PNI ≤ 38.6 were divided into a high PNI group and a low PNI group^16,17^.

**Table 1:** Evaluation of the nourishing status utilizing the CONUT score.

Factor	None	Light	Moderate	Severe
Serum albumin (g/dL)	> 3.50	3.49–3.0	2.50–2.99	< 2.5
Score	0	2	4	6
Total lymphocyte count (/mm^3^)	> 1600	1200–1599	800–1199	< 800
Score	0	1	2	3
Total cholesterol (mg/dL)	> 180	140–179	100–139	< 100
Score	0	1	2	3
Total Score	0	4	8	12

### Clinical cure standard

(1) Clinical symptoms and signs related to spinal tuberculosis disappeared for more than 3 months; (2) different degrees of recovery of neurological dysfunction; (3) local spinal tuberculosis showed no signs of infection, and the sinus had healed without exudation; (4) ESR and CRP were normal for three consecutive times; and (5) imaging examination revealed no abscess, dead bone or fusion with bone graft^18^.

### Postoperative management and evaluations

The combination of antituberculous consisting of isoniazid (5 mg/kg), rifampicin (10 mg/kg), ethambutol (15 mg/kg), and pyrazinamide (25 mg/kg) was administered for 2 months after the operations. Thereafter, a regimen of rifampicin, isoniazid, and ethambutol was administered for at least 10 months. Patients were followed up every month in the first three months after surgery, and then every 3–6 months. Follow-up was conducted by telephone, outpatient service and medical records. At the postoperative follow-up of 1 year, according to the clinical cure standards, the patients were divided into an effective prognosis group and an ineffective prognosis group. Patients who did not reach the clinical cure standard at the end point continued anti-tuberculosis treatment. X-ray, construction of computer tomography (CT) and magnetic resonance imaging (MRI) of the surgical site were reviewed at 3, 6 and 12 months postoperatively to determine the occurrence of bone graft fusion and internal fixation loosening, the condition of deformity correction and the presence paravertebral abscess. Postoperative complications were observed and recorded: sinus formation, no fusion of bone graft, pneumonia, deep vein thrombosis, low urinary tract infection, liver injury, incision infection, etc.

### Statistical analysis

The results of the descriptive statistical analysis were expressed as the mean ± standard deviation (SD) when they were continuous data and the number (N) and percentage (%) when they were categorical data. Fisher's exact (chi-squared) test or the χ^2^ test was used to compare categorical data, and the Mann–Whitney U test or independent sample T test was used to compare continuous data. Receiver operating characteristic (ROC) curves were plotted, the area under the curve (AUC) was used to evaluate sensitivity and specificity, and the Youden index was estimated to determine the best cut-off value of PNI. The AUCs were compared using the DeLong method. Spearman’s test was used to evaluate the correlation between the PNI and CONUT score. The independent predictors of STB were determined by univariate and multivariate logistic regression. Odds ratios (ORs) and 95% confidence intervals (CIs) were calculated. P < 0.05 was considered statistically significant. SPSS Software Version 26.0 and Medcalc 20.0 were used for the statistical analysis.

### Ethics approval and consent to participate

This study has been approved by the Ethics Committee of the Second Affiliated Hospital of Chongqing Medical University. The committee’s reference number: 2022 Ethics Review No. 6.

## Results

### Baseline characteristics

The basic characteristics of patients at initial treatment are shown in Table 2. The mean age was 53.9 years (range from 19 to 82 years), and the mean BMI was 20.5 kg/m^2^. Fifty-two patients (53.6%) were male. Nine patients (9.3%) and five patients (5.2%) had a history of hypertension and diabetes, respectively. The included patients were divided into the high PNI and low PNI groups based on the PNI cut-off value of 38.6, and 71 patients (73.2%) were classified into the high PNI group. The age of the high PNI group was significantly lower than that of the low PNI group, the BMI of the high PNI group was significantly better than that of the low PNI group, and the patients in the low PNI group were more likely to have pneumonia and lower urinary tract infection than the patients in the high PNI group (Table 2). The included patients were also divided into the low CONUT group and the high CONUT group according to the CONUT cut-off value of 5, and their clinical characteristics were similar to those of the PNI group (Table 3). There was an obvious correlation between the PNI and CONUT score according to Spearman’s correlation analysis (r =  − 0.884, P < 0.05).

**Table 2:** Characteristic in patients with different PNI.

Characteristic	Total (n = 97)	PNI		*p* value
		Low-PNI (≤ 38.6), (n = 26)	High-PNI (> 38.6), (n = 71)	
Age (years old), mean ± SD	53.9 ± 17.1	64.9 ± 11.5	49.9 ± 17.0	< 0.001^c^
Gender (male, %)	52 (53.6)	16 (61.5)	36 (50.7)	0.343^a^
BMI (kg/m^2^), mean ± SD	20.5 ± 3.1	18.6 ± 2.5	21.1 ± 3.0	< 0.001^c^
Duration of illness (> 6 months), n (%)	48 (49.5)	14 (53.8)	34 (47.9)	0.603^a^
Duration of anti-TB agents (month), mean ± SD	12.5 ± 1.0	13.5 ± 1.4	12.1 ± 0.4	< 0.001^c^
Hypertension, n (%)	9 (9.3)	4 (15.4)	5 (7.0)	0.243^b^
Diabetes mellitus, n (%)	5 (5.2)	1 (3.8)	4 (5.6)	1.000^b^
Cardiovascular disease, n (%)	3 (3.1)	1 (3.8)	2 (2.8)	1.000^b^
Smoking, n (%)	23 (23.7)	8 (30.8)	15 (21.1)	0.323^a^
Drinking, n (%)	21 (21.6)	7 (26.9)	14 (19.7)	0.445^a^
**Vertebral body involvement**				
Abscess, n (%)	64 (66.0)	17 (65.4)	47 (66.2)	0.940^a^
Kyphotic deformity, n (%)	9 (9.3)	1 (3.8)	8 (11.3)	0.437^b^
Vertebra collapse, n (%)	32 (33.0)	10 (38.5)	22 (31.0)	0.488^a^
Bone destruction, n (%)	89 (91.8)	24 (92.3)	65 (91.5)	1.000^b^
Cord compression, n (%)	40 (41.2)	12 (46.2)	28 (39.4)	0.552^a^
Involvement of > 2 vertebrae, n (%)	34 (35.1)	10 (38.5)	24 (33.8)	0.670^a^
Location of junction lesions, n (%)	29 (29.9)	11 (42.3)	18 (25.4)	0.106^a^
Extra-osseous lesions, n (%)	31 (32.0)	12 (46.2)	19 (26.8)	0.070^a^
Neurological dysfunction, n (%)	38 (39.2)	12 (46.2)	26 (36.6)	0.394^a^
**Complications**				
Pneumonia, n (%)	18 (18.6)	13 (50.0)	5 (7.0)	< 0.001^b^
Sinus tract, n (%)	5 (5.2)	3 (11.5)	2 (2.8)	0.118^b^
Incision infection, n (%)	5 (5.2)	3 (11.5)	2 (2.8)	0.118^b^
Deep vein thrombosis, n (%)	2 (2.1)	1 (3.8)	1 (1.4)	0.466^b^
Bone graft non fusion, n (%)	1 (1.0)	0 (0)	1 (1.4)	1.000^b^
Liver injury, n (%)	3 (3.1)	2 (7.7)	1 (1.4)	0.174^b^
Lower urinary tract infection, n (%)	6 (6.2)	4 (15.4)	2 (2.8)	0.043^b^
Renal failure, n (%)	1 (1.0)	1 (3.8)	0 (0)	0.268^b^

*PNI* prognostic nutrition index, *BMI* body mass index, *SD* standard deviation.

^a^χ^2^ test.

^b^Fisher’s exact test.

^c^T-test.

**Table 3:** Characteristic in patients with different CONUT.

Characteristic	Total (n = 97)	CONUT		p value
		Low-CONUT (≤ 5), (n = 61)	High-CONUT (> 5), (n = 36)	
Age (years old), mean ± SD	53.9 ± 17.1	49.6 ± 17.4	61.2 ± 13.9	0.001^c^
Gender (male, %)	52 (53.6)	31 (50.8)	21 (58.3)	0.043^a^
BMI (kg/m^2^), mean ± SD	20.5 ± 3.1	21.6 ± 2.9	18.5 ± 2.5	< 0.001^c^
Duration of illness (> 6 months), n (%)	48 (49.5)	32 (52.5)	16 (44.4)	0.446^a^
Duration of anti-TB agents (month), mean ± SD	12.5 ± 1.0	12.1 ± 0.3	13.2 ± 1.4	< 0.001^c^
Hypertension, n (%)	9 (9.3)	5 (8.2)	4 (11.1)	0.723^b^
Diabetes mellitus, n (%)	5 (5.2)	4 (6.6)	1 (2.8)	0.648^b^
Cardiovascular disease, n (%)	3 (3.1)	2 (3.3)	1 (2.8)	1.000^b^
Smoking, n (%)	23 (23.7)	13 (21.3)	10 (27.8)	0.469^a^
Drinking, n (%)	21 (21.6)	9 (14.8)	12 (33.3)	0.032^a^
**Vertebral body involvement**				
Abscess, n (%)	64 (66.0)	40 (65.6)	24 (66.7)	0.913^a^
Kyphotic deformity, n (%)	9 (9.3)	5 (8.2)	4 (11.1)	0.723^b^
Vertebra collapse, n (%)	32 (33.0)	16 (26.2)	16 (44.4)	0.065^a^
Bone destruction, n (%)	89 (91.8)	55 (90.2)	34 (94.4)	0.706^b^
Cord compression, n (%)	40 (41.2)	23 (37.7)	17 (47.2)	0.358^a^
Involvement of > 2 vertebrae, n (%)	34 (35.1)	20 (32.8)	14 (38.9)	0.543^a^
Location of junction lesions, n (%)	29 (29.9)	17 (27.9)	12 (33.3)	0.570^a^
Extra-osseous lesions, n (%)	31 (32.0)	16 (26.2)	15 (41.7)	0.115^a^
Neurological dysfunction, n (%)	38 (39.2)	23 (37.7)	15 (41.7)	0.699^a^
**Complications**				
Pneumonia, n (%)	18 (18.6)	4 (6.6)	14 (38.9)	< 0.001^a^
Sinus tract, n (%)	5 (5.2)	2 (3.3)	3 (8.3)	0.357^b^
Incision infection, n (%)	5 (5.2)	2 (3.3)	3 (8.3)	0.357^b^
Deep vein thrombosis, n (%)	2 (2.1)	1 (1.6)	1 (2.8)	1.000^b^
Bone graft non fusion, n (%)	1 (1.0)	0 (0)	1 (2.8)	0.371^b^
Liver injury, n (%)	3 (3.1)	1 (1.6)	2 (5.6)	0.553^b^
Lower urinary tract infection, n (%)	6 (6.2)	2 (3.3)	4 (11.1)	0.191^b^
Renal failure, n (%)	1 (1.0)	0 (0)	1 (2.8)	0.371^b^

*CONUT* controlling nutritional status, *BMI* body mass index, *SD* standard deviation.

^a^χ^2^ test.

^b^Fisher’s exact test.

^c^Independent Sample T-test.

**Figure 1:**
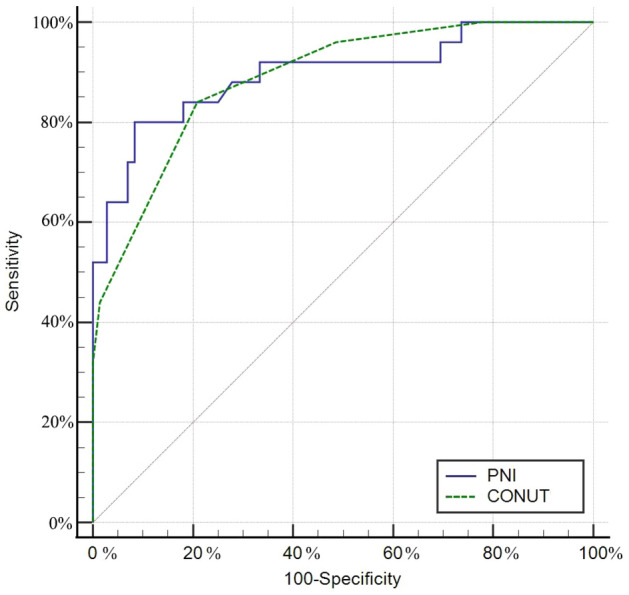
Receiver operating characteristic analysis of PNI and CONUT score in predicting prognosis of STB postoperatively. *CONUT* controlling nutritional status, *PNI* prognostic nutrition index, *STB* spinal tuberculosis.

The ROC curve of unfavourable outcomes showed that the CONUT score and PNI had predictive value. The CONUT score predicted unfavourable prognosis with an AUC of 0.888 (95% CI 0.808–0.943, P < 0.001), similar to a PNI of 0.896 (95% CI 0.818–0.949, P < 0.001) (Fig. 1). The sensitivity and specificity of expected performance were 84.0% and 79.17% for the CONUT score and 80.0% and 91.67% for the PNI, respectively. The De Long method showed no significant difference in AUC between the CONUT and PNI (P = 0.710).

### Effect of the CONUT score and PNI on prognosis

Seventy-two (74.2%) patients in our research had an effective result at one year, among which 57 (79.2%) of them had a low CONUT score, and 66 (91.7%) of them had a high PNI. The low COUNT and high PNI groups mostly overlapped. The correlation between clinical prognosis and basic clinical characteristics were explored by a univariate analysis one year postoperatively. The results showed that patients in the unfavourable group were older and had a higher rate of pneumonia and lower urinary tract infection, a lower PNI and a higher CONUT score than those in the favourable group (P < 0.05) (Table 4). In the multivariate analysis of unfavourable outcomes, the multiple regression established two models, both of which included CONUT or PNI. The multivariate analysis showed that the CONUT score and PNI were two independent predictors of unfavourable STB outcomes (Table 5), and the adjusted ORs were 2.447 (95% CI 1.518–4.043, P < 0.001) and 0.689 (95% CI 0.563–0.843, P < 0.001), respectively.

**Table 4:** Univariate analysis of included patients by one-year clinical prognosis in general.

Factors	Effective clinical prognosis (n = 72)	Ineffective clinical prognosis (n = 25)	Exp (B)	95%CI	*p* value
Age (years old), mean ± SD	50.7 ± 17.3	63.1 ± 12.6	1.054	1.018–1.091	0.003
Gender (male, %)	35 (48.6)	17 (68.0)	0.445	0.171–1.161	0.098
BMI (kg/m^2^), mean ± SD	20.8 ± 2.9	19.4 ± 3.4	0.852	0.725–1.001	0.052
Duration of illness (> 6 months), n (%)	37 (51.4)	11 (44.0)	0.743	0.298–1.856	0.525
Hypertension, n (%)	5 (6.9)	4 (16.0)	2.552	0.627–10.383	0.191
Diabetes mellitus, n (%)	4 (5.6)	1 (4.0)	0.708	0.075–6.655	0.763
Cardiovascular disease, n (%)	2 (2.8)	1 (4.0)	1.458	0.126–16.812	0.762
Smoking, n (%)	15 (20.8)	8 (32.0)	1.788	0.648–4.933	0.262
Drinking, n (%)	16 (22.2)	5 (20.0)	0.875	0.284–2.699	0.816
**Vertebral body involvement**					
Abscess, n (%)	49 (68.1)	15 (60.0)	0.704	0.275–1.804	0.465
Kyphotic deformity, n (%)	7 (9.7)	2 (8.0)	0.807	0.156–4.170	0.798
Vertebra collapse, n (%)	22 (30.6)	10 (40.0)	1.515	0.589–3.895	0.388
Bone destruction, n (%)	66 (91.7)	23 (92.0)	1.045	0.197–5.549	0.958
Cord compression, n (%)	30 (41.7)	10 (40.0)	0.933	0.369–2.359	0.884
Involvement of > 2 vertebrae, n (%)	25 (34.7)	9 (36.0)	1.057	0.409–2.734	0.908
Location of junction lesions, n (%)	21 (29.2)	8 (32.0)	1.143	0.428–3.051	0.790
Extra-osseous lesions, n (%)	18 (25.0)	13 (52.0)	3.250	1.258–8.393	0.015
Neurological dysfunction, n (%)	27 (37.5)	11 (44.0)	1.310	0.520–3.295	0.567
**Complications**					
Pneumonia, n (%)	5 (6.9)	13 (52.0)	14.517	4.370–48.222	< 0.001
Sinus tract, n (%)	2 (2.8)	3 (12.0)	4.773	0.749–30.421	0.098
Incision infection, n (%)	3 (4.2)	2 (8.0)	2.000	0.314–12.724	0.463
Deep vein thrombosis, n (%)	2 (2.8)	0 (0)	< *0.001*	NA	0.999
Bone graft non fusion, n (%)	0 (0)	1 (4.0)	NA	NA	1.000
Liver injury, n (%)	1 (1.4)	2 (8.0)	6.174	0.535–71.266	0.145
Lower urinary tract infection, n (%)	2 (2.8)	4 (16.0)	6.667	1.140–38.984	0.035
Renal failure, n (%)	0 (0)	1 (4.0)	NA	NA	1.000
Serum albumin (g/dL), mean ± SD	3.8 ± 0.4	3.1 ± 0.5	0.017	0.003–0.097	< 0.001
Total lymphocytes (/mL),	1263.6 ± 475.5	967.2 ± 449.5	0.999	0.997–1.000	0.012
Total cholesterol (mg/dL),	90.3 ± 17.4	82.7 ± 19.0	0.977	0.953–1.003	0.078
CONUT score, n (%)			0.644	0.532–0.781	< 0.001
Low-CONUT (≤ 5)	57 (79.2)	4 (16.0)			
High-CONUT (> 5)	15 (20.8)	21 (84.0)			
PNI, n (%)			2.957	1.871–4.673	< 0.001
Low-PNI (≤ 38.6)	6 (8.3)	20 (80.0)			
High-PNI (> 38.6)	66 (91.7)	5 (20.0)			

*CONUT* controlling nutritional status, *PNI* prognostic nutrition index, *BMI* body mass index, *SD* standard deviation, *95%CI* 95% confidence interval, *NA* not available.

**Table 5:** Results of multivariate logistic analysis of the risk factors in participating patients with poor utilitarian results at one year.

Risk factors	β	OR	95%CI	p value
**Model 1**				
Age, per 1 y	0.001	1.001	0.958–1.047	0.958
Extra-osseous lesions, present vs absent	0.815	2.258	0.542–9.408	0.263
Pneumonia, present vs absent	1.703	5.488	1.069–28.185	0.041
Lower urinary tract infection, present vs absent	1.627	5.088	0.203–127.748	0.323
PNI, per 1 score	− 0.373	0.689	0.563–0.843	< 0.001
**Model 2**				
Age, per 1 y	0.010	1.010	0.965–1.057	0.670
Extra-osseous lesions, present vs absent	0.820	2.271	0.551–9.364	0.256
Pneumonia, present vs absent	1.828	6.222	1.227–31.555	0.027
Lower urinary tract infection, present vs absent	1.771	5.875	0.209–165.167	0.298
CONUT score, per 1 score	0.907	2.477	1.518–4.043	< 0.001

*CONUT* controlling nutritional status, *PNI* prognostic nutrition index, *95%CI* 95% confidence interval, *OR* odds ratios.

## Discussion

The purpose of the present study was to analyse the relationship between PNI, CONUT score and prognosis after spinal tuberculosis surgery and to determine the risk factors for complications. In this single-centre, retrospective study, our study showed that patients with a higher CONUT score and lower PNI had poorer outcomes than patients with a lower CONUT score and higher PNI at the 1-year follow-up. The multivariate analysis showed that the CONUT score and PNI were independent predictors of adverse outcomes of spinal tuberculosis postoperatively.

Globally, tuberculosis is the second most fatal disease caused by a single source of infection. Spinal tuberculosis (STB) is usually haematogenously diffused into vertebral cancellous bone by Mycobacterium tuberculosis from lesions in the lung or urogenital system via venous or arterial routes. At different disease sites of tuberculosis, multiple mycobacteria have different growth kinetics and metabolic characteristics. Organs with high tissue oxygen content, such as the lung, a tend to involve multiple bacteria. However, in closed bone tissue, there are more dormant mycobacteria, which are difficult to kill^19^. Therefore, simple drug therapy needs to rely on multidrug combinations and standardized and adequate treatment to reduce recurrence and drug resistance. For spinal tuberculosis patients without neurological defects and spinal deformities, the prognosis is generally good. However, surgical debridement and internal fixation should be performed for patients with confirmed or predicted spinal malformations, neurological defects, or paravertebral abscesses or for patients who cannot be diagnosed by percutaneous biopsy. Park DW et al.^20^ indicated that radical surgery is significantly associated with a better prognosis.

Tuberculosis exists in two forms: latent and active. When Mycobacterium tuberculosis reaches the host, it activates the immune response, but this is not enough to eliminate Mycobacterium tuberculosis. Most infected people are in a state of latent tuberculosis. When patients suffer from malnutrition and cellular immune function declines, tuberculosis reactivation will occur and progress to active tuberculosis. However, with the improvement of nutritional status, the damage to immune function can be improved, and the incidence rate can be reduced. Active TB, like other infectious diseases, carries a high energy expenditure and therefore requires an additional 20–30% of daily intake^6,21^. Therefore, malnutrition is considered to be a widespread risk factor for TB. Nutrition and infection interact with each other synergistically. Repeated infection leads to the loss of nitrogen in the body and the deterioration of nutritional status. The resulting malnutrition in turn makes people more susceptible to infection. Malnutrition not only worsens tuberculosis but is also a risk factor for drug poisoning and death during treatment^22^.

The immune protection mechanism of tuberculosis depends most on the interaction and synergy between T cells, monocytes-macrophages and nutrition-sensitive cytokines^23–25^. As the interstitium between the innate immune system and adaptive immune system, dendritic cells present antigens to T cells and B cells once activated. Circulating monocytes can produce proinflammatory cytokines, such as tumour necrosis factor α (TNF-α), to stimulate the inflammatory response to pathogens. TNF-α initiates a series of events through tumour necrosis factor receptor 1 (TNFR1), including activation of nuclear factor-κB (NF-κB) and promotion of cytokine and chemokine production^26,27^. Malnutrition has a strong relationship with a series of impaired immune functions. Malnutrition in the experimental model significantly reduces dendritic cells and proinflammatory cytokines. At the same time, macrophages lacking protein produce more transforming growth factor β, thereby inhibiting the function of T lymphocytes in infected animals^28,29^.

Nutritional status assessment is divided into subjective evaluation and objective evaluation. Subjective evaluations include the body function scale reflected in nutrition, such as the patient-generated subjective global assessment (PG-SGA) and the Malnutrition Universal Screening Tool (MUST). Lin H.S. et al. and Miyata S. et al.^30,31^ used PG-SGA and MUST, respectively, to assess malnutrition and noted that the assessment scale was significantly associated with tuberculosis prognosis and could be used as a prognostic indicator for tuberculosis patients. However, these scales contain a variety of subjective factors and cannot be accurately evaluated clinically. Objective criteria include laboratory tests and anthropometric assessments such as body mass index (BMI), weight change, and measured immunity, although Bhargava M et al.^32^ proposed a simplified chart based on BMI to assess the nutritional status of TB patients. However, for a large number of elderly patients, obsolete spine fractures, scoliosis, kyphosis, leg bending, and flattening of the plantar arch may lead to inaccurate height measurements and overestimation of BMI. BMI is also of no significance in predicting fatal results. A single measurement method lacks sensitivity and specificity and cannot be used as a reliable nutritional status indicator. Yu-Cheng Bao et al.^10^ proposed that the change in prealbumin in perioperative blood is consistent with improvements in nourishment and inflammation and is related to the rate of incision infection. Kim and Sudprasert et al.^11,12^ analysed the prognosis of spinal tuberculosis using the erythrocyte sedimentation rate (ESR) and C-reactive protein (CRP ), respectively, and concluded that the decrease in ESR and CRP in the early postoperative period was helpful for the prognosis of spinal tuberculosis and the recovery of neurological function. However, that conclusion is limited to the results of a single laboratory examination and fails to fully assess the nutritional and immune status of patients.

The CONUT score and PNI are immune-nutritional screening tools consisting of different immune and nutritional biomarkers. PNI consists of two laboratory indicators, albumin and lymphocyte count. CONUT is divided into three parts, including nutritional indicators of serum albumin and immune status indicators of cholesterol and lymphocyte count. Serum albumin can reflect the nutritional status, and serum lymphocyte count has been identified as an objective parameter of inflammation. The ratio of neutrophils to lymphocytes is related to the prognosis of tuberculosis^33–35^. In addition to its central role in lipid storage, adipose tissue has a major endocrine function and releases a variety of proinflammatory and anti-inflammatory factors, such as leptin, adiponectin and resistance, as well as cytokines and chemokines. Changes in adipocytokine levels have been observed under various inflammatory conditions. Leptin, in particular, promotes the proliferation and activation of T lymphocytes under mitogen stimulation, playing an important role in immune response and inflammation. Our study shows that the CONUT score and PNI can predict adverse outcomes in patients with spinal tuberculosis postoperatively^36,37^.

Based on our study, the PNI and CONUT score can guide the perioperative treatment and nursing of spinal tuberculosis and provide personalized treatment. These prognostic indicators of nutritional status are very important for assessing patients' perioperative status and are combined with clinical treatment guidelines to minimize the decline in nutritional immune status of patients with spinal tuberculosis in the early stage of treatment and promote early postoperative rehabilitation and functional exercise. For patients with gastrointestinal dysfunction, parenteral nutrition should also be provided as early as possible to provide targeted calories.

The limitations of our study are as follows: First, this study is a retrospective, single-centre study with a small sample size. Second, we assessed only the preoperative nutritional status of patients and not the postoperative nutritional status. Further prospective studies are needed to evaluate the relationship between the PNI, CONUT score and prognosis of patients with spinal tuberculosis. In addition, many studies note that trace elements potentially influence TB infection and immunity, but the measurement of trace elements was not included in this study. Because the contents of vitamins and trace elements are very low, technology for the analysis of these nutrients should be sufficiently sensitive, and caution must be used when measuring such compounds to avoid pollution.

## Conclusion

In conclusion, higher CONUT scores and a lower PNI were associated with adverse outcomes in patients with spinal tuberculosis. The CONUT score and PNI are inexpensive and readily available biomarkers that may help identify patients with unfavourable prognoses who would benefit from early nutritional therapy. These findings should be evaluated in further prospective studies.

## Data Availability

The datasets used and/or analysed during the current study are available from the corresponding author on reasonable request.

## Abbreviations

PNI: Prognostic Nutritional Index
CONUT: Controlling nutritional status
BMI: Body mass index
ROC: Receiver operating characteristic
CT: Computer tomography
MRI: Magnetic resonance imaging
SD: Standard deviation
AUC: Area under the curve
OR: Odds ratios
CI: Confidence intervals
HIV: Human immunodeficiency infection
STB: Spinal tuberculosis
WHO: World Health Organization
IFN: Interferon
Th1: Type 1 T-assisted
TNF: Tumor necrosis factor
TNFR1: Tumor necrosis factor receptor 1
NF: Nuclear factor
PG-SGA: Patient-generated subjective global assessment
MUST: Malnutrition Universal Screening Tool
ESR: Erythrocyte sedimentation rate
CRP: C-reactive protein
